# Medical Confidences and the Courts of Law

**Published:** 1906-12-01

**Authors:** 


**The Hospital**

A JOURNAL OF

The Medical Sciences and Hospital Administration.

VOL. XLI.—No. 1,054. SATURDAY, DECEMBER 1, 1906.

### MEDICAL CONFIDENCES AND THE COURTS OF LAW.

THE vexed and troublesome question of a medical man's liability to be compelled, in a court of law, to disclose information which he has gained from a patient under the usual conditions of professional secrecy, receives further illustration in an incident recently reported from the Sheriff Court of Glasgow. The event is all the more important in view of the fact that, and in opposition to many previous precedents, the presiding judge declined to put pressure on the medical witness and upheld his objection to reveal the secrets of his patient. The question arose in the course of a civil action in which, among the witnesses summoned, was Dr. D. C. McVail, one of the physicians to the Glasgow Royal Infirmary. Dr. McVail's knowledge of the point at issue was derived solely from the fact that one of the parties to the dispute had been a patient under his care in the hospital, and he was called by the opposite side to prove, presumably, something to their opponent's discredit. He was thus asked to give evidence based solely and entirely upon the private professional relationships which had existed between himself and his patient. Upon entering the witness-box Dr. McVail at once claimed that, in the circumstances, he ought not to be sworn or to be asked questions which he could only answer by an abuse of his patient's confidence. The solicitor representing the interests of the person by whom Dr. McVail's evidence was desired protested against this claim; but, after hearing arguments, the presiding judge, Sheriff Davidson, sustained Dr. McVail's objection and dismissed him from the case.

The decision of the Sheriff on this point will, we think, come as a surprise both to lawyers and to medical men, though it will probably be much more welcome to the latter than to the former. It is certainly in conflict with many previous legal rulings on the same point, and with the teaching provided for medical students in the current manuals of forensic medicine. Indeed, in these it is usually laid down, as a matter entirely beyond dispute, that a medical witness can claim no privilege in this respect in a court of law—that, in short, a plea of professional confidence is not a valid basis on which to rest a refusal to answer a question allowed by the Court. In the case above alluded to the Court refused to allow any questions to be put to the witness, and, for aught we know, this decision may be subject to review by some higher authority. Further, there may be other technical aspects of the decision which can only be appreciated by members of the legal profession. It must also be noted that the Court in which the point was raised and decided is one of summary jurisdiction, and therefore it by no means follows that the decision can be successfully quoted as a precedent in other instances; indeed, there are not a few decisions of the superior Courts in an entirely opposite direction. Still, the fact remains that a medical witness has in a public trial and by a recognised judicial authority been excused from giving evidence on the ground that such evidence would be a betrayal of the trust and confidence placed in him in his professional capacity by one of the parties to the case. We think Dr. McVail is to be congratulated on this result, which is the direct outcome of his sense of professional duty and public spirit, and whatever be the effect on legal practice, the moral value of the decision he has secured cannot fail to be a considerable one.

There can be no doubt that the medical profession entertains a decided objection to the compulsory disclosure of professional confidences in the courts of law; and this more in the public interests than for any selfish reason. Hitherto, at least for the most part, the judges have refused to pay any attention to this objection. Yet it is possible in recent years to trace some modification of this extreme legal position. Prominent in this respect is the statement of Mr. Justice Hawkins, in the case of Kitson *v.* Playfair, that he "could quite understand a case, especially a civil cause, where a doctor was quite justified in refusing to divulge questions of professional secrecy." Some few years ago, too, the magisterial bench at Nottingham sustained the protest of a medical man who declined to give evidence regarding the nature of an illness from which one of his patients suffered whilst living apart from her husband. The opposing solicitor demanded that the medical witness should be committed to prison, but the magistrates refused to take action. To these may now be added the decision of

Sheriff Davidson on the protest of Dr. McVail. Such a series of facts suggests that the legal authorities may, under sustained pressure, be compelled to modify the high-and-dry doctrine in accordance with which they have hitherto been in the habit of forcing the professional confidences of a medical witness. Certainly these decisions lend force to the advice that medical practitioners should not quietly and without protest acquiesce in any such demand. If the Court refuses to sustain the protest, the witness must, of course, either yield or go to prison. But in either case it is made evident to all who may be concerned that the responsibility for the disclosure rests with the law and not with the habit of the medical profession. That habit is one of unbroken respect for all professional confidences, and if this is to be invaded the responsibility for the invasion must be sustained by those who direct the legal procedure of the country. Let medical witnesses continue the good example set by Dr. McVail, and we fancy it will not be long before the public recognises that the best interests of the community require that medical confidences shall be secure even from the wiles and suggestions of legal cross-examination.

There is one further point in the case here discussed. It is provided by the fact that the person concerned was a hospital patient. The medical profession will not for a moment allow that any difference exists in this matter between hospital patients on the one hand and private patients on the other. But it may be fairly questioned whether solicitors fully understand this attitude. It is not a very uncommon thing for members of the visiting staffs of hospitals or for resident medical officers to receive requests from solicitors for information regarding patients who are or have been in hospital. All such requests, we hold, should be firmly refused unless fully endorsed by the patient concerned. Maintenance of this attitude by all members of the profession will be a substantial help towards impressing on the legal authorities an appreciation of the impartial respect with which members of the medical profession treat the confidences of their patients.

